# FcgRIIIA (CD16)-expressing monocytes mediate the depletion of tumor-infiltrating Tregs via Ipilimumab-dependent ADCC in melanoma patients

**DOI:** 10.1186/2051-1426-2-S3-O14

**Published:** 2014-11-06

**Authors:** Emanuela Romano, Monika Kusio-Kobialka, Periklis G Foukas, Helene Bichat, Petra Baumgaertner, Christiane Meyer, Pierluigi Ballabeni, Olivier Michielin, Benjamin Weide, Pedro Romero, Daniel E Speiser

**Affiliations:** 1 CHUV Service of Oncology and Ludwig Cancer Research Center, Department of Oncology, Lausanne, Switzerland; 2 CHUV, Department of Oncology, Lausanne, Switzerland; 3 Ludwig Cancer Research Center, Department of Oncology, Lausanne, Switzerland; 4 CHUV and Ludwig Cancer Research Center, Department of Oncology, Lausanne, Switzerland; 5 Institute of Social and Preventive Medicine (IUMSP), Lausanne, Switzerland; 6 Division of Dermatooncology, Dept. of Dermatology, University Medical Center, Tuebingen, Germany; 7 Ludwig Cancer Research Center, Lausanne, Switzerland

## 

Enhancing immune responses with immune-modulatory monoclonal antibodies (mAbs) directed to inhibitory immune-receptors is a promising modality in cancer therapy. Clinical efficacy has been demonstrated with antibodies blocking inhibitory immune checkpoints such as CTLA-4 or PD-1/PD-L1. Treatment with Ipilimumab (Yervoy^®^), a fully human CTLA-4 specific mAb, showed durable clinical efficacy and improved overall survival in metastatic melanoma; its mechanism(s) of action, however, are only partially understood. Recent studies in melanoma mouse models revealed that the anti-tumor activity of CTLA-4 blockade is mediated by FcgRIV-expressing macrophages in the tumor microenvironment (TME) via *in-trans *depletion of tumor-infiltrating regulatory T cells (Tregs). We speculated that a similar mechanism might operate in melanoma patients responding to Ipilimumab. To investigate this hypothesis, we interrogated peripheral blood mononuclear cells (PBMCs) and matched melanoma metastases from 15 patients responding (R) and 14 non-responding (NR) to Ipilimumab. Our findings show, for the first time, that Ipilimumab leads to the depletion *in vitro *of Tregs via an antibody-dependent-cellular-cytotoxicity (ADCC) mechanism, selectively mediated by FcgRIIIA (CD16)-expressing, nonclassical monocytes (CD14^+^CD16^++^). In contrast, classical CD14^++^CD16^- ^monocytes, lacking the FcgRIIIA expression, are unable to deplete Tregs in an ADCC assay. Interestingly, patients responding to Ipilimumab displayed significantly higher baseline peripheral frequencies of nonclassical monocytes than nonresponder patients. Evaluation of matched melanoma metastases from pre- and post-Ipilimumab time-points by IHC revealed that, in the TME, responders had the highest CD68^+^/CD163^+ ^macrophage ratios at baseline, and showed decreased infiltration of Tregs after treatment. Notably, baseline Treg infiltration was comparable between the two groups. Our findings provide novel mechanistic insight into the clinical activity of Ipilimumab, highlighting the contribution of host-dependent factors into the final outcome of antibody-based immune-modulatory therapies and identify nonclassical monocytes as a potential biomarker of response.

## Consent

Written informed consent was obtained from the patient for publication of this abstract and any accompanying images. A copy of the written consent is available for review by the Editor of this journal.

